# Mesoscopic simulations of active nematics

**DOI:** 10.1126/sciadv.abo5788

**Published:** 2022-08-24

**Authors:** Timofey Kozhukhov, Tyler N. Shendruk

**Affiliations:** School of Physics and Astronomy, The University of Edinburgh, Peter Guthrie Tait Road, Edinburgh EH9 3FD, UK.

## Abstract

Coarse-grained, mesoscale simulations are invaluable for studying soft condensed matter because of their ability to model systems in which a background solvent plays a substantial role but is not the primary interest. Such methods generally model passive solvents; however, far-from-equilibrium systems may also be composed of complex solutes suspended in an active fluid. Yet, few coarse-grained simulation methods exist to model an active medium. We introduce an algorithm to simulate active nematics, which builds on multiparticle collision dynamics (MPCD) for passive fluctuating nematohydrodynamics by introducing dipolar activity in the local collision operator. Active nematic MPCD (AN-MPCD) simulations not only exhibit the key characteristics of active nematic turbulence but, as a particle-based algorithm, also reproduce crucial attributes of active particle models. Thus, mesoscopic AN-MPCD is an approach that bridges microscopic and continuum descriptions, allowing simulations of composite active-passive systems.

## Teaser

Mesoscale simulations of active nematics bridge the gap between micro- and macroscopic active systems.

## INTRODUCTION

While fish shoals, bird flocks, and insect swarms are magnificent macroscopic examples of active systems that captivate onlookers with their collective behaviors (*1*), the majority of biophysical research focuses on active systems composed of microscopic agents. Microscopic, motile particles locally convert free energy from their surroundings into mechanical work and collective dynamics (*2*, *3*). These active particles are subject to relatively large stochastic fluctuations that play a substantial role in their dynamics. Prominent examples of active stochastic systems include suspensions of swimming microbes (*4*), bacterial colonies (*5*), tissue monolayers (*6*, *7*), and mixtures of cytoskeletal filaments and motor proteins (*8*).

In addition to having microscopic activity and stochastic dynamics, each of these examples displays orientational ordering (*9*–*11*). While shape anisotropy is not a strict prerequisite of active self-propulsion, an innate direction of self-propulsion typically is. Furthermore, directionality regularly materializes as apolar orientation, even when the microscopic agents have polar motility (*12*). Despite the fact that swimming bacteria (*10*), kinesin motor proteins marching along microtubules (*8*), and most other constituent self-propelled particles (*3*) have distinctly polar behavior, the hydrodynamic limit of suspensions of many such particles is nematic in nature. This is because the interactions—including dipolar forces enacted on the surrounding fluid medium—are principally apolar (*13*). Thus, active nematics have proven to be a fruitful model for studying intrinsically out-of-equilibrium materials.

Alongside biophysical experiments, numerical simulations of nematic systems have been essential in developing a physical understanding of activity’s consequences for living materials. Different studies have attempted to model the microscopic details to a greater or lesser extent. For example, simulations of simple self-propelled rods in the dry limit (*12*) have found many of the same properties as more detailed simulations of substrate-crawling bacilliforms (*14*). Similarly, self-propelled rods in the wet limit (*15*) agree with simulations of swimming bacteria (*16*). While the microscopic details of active agents can vary notably often, even simple models can reproduce the essential emergent behaviors (*3*). Simple microscopic models of stochastic, self-propelled particles (such as nematic variations on the Vicsek model) (*17*, *18*) have been particularly well studied and can be coarse-grained into kinetic theories, which, in turn, lead to specific hydrodynamic equations of motion (*19*).

On the other hand, symmetry considerations allow one to write generalized hydrodynamic equations for active fluids in the continuum limit (*2*, *20*). Unlike the microscopic models, these often omit stochastic effects within the fluid. Substantial work has explored the physics of bulk active fluids, such as steady-state creation and annihilation of topological defects (*21*, *22*) and the emergence of low–Reynolds number active turbulence (*23*–*26*). The emergence of complex spontaneous flows and topological singularities from field equations suggests that confining active nematics could produce complex and dynamic self-actualized dissipative structures. However, studies of confined active nematics have mostly been limited to simple, fixed geometries (*27*–*31*), and more complicated microfluidic geometries have rarely been considered.

Similarly, passive solutes suspended in active fluids have not received extensive consideration (*20*, *32*). Driven particles, such as colloids and disks within active nematics, have exhibited exciting characteristics, such as effective negative viscosity (*33*) and higher-order defects (*34*). Experimentally, particles embedded in dense suspensions of swimming bacteria have exhibited anomalous diffusion (*20*, *35*). While polymers suspended in intrinsically out-of-equilibrium athermal baths have been studied (*36*–*38*), hydrodynamic interactions mediated through the active medium are typically neglected (*39*). This highlights the need for coarse-grained simulation techniques capable of simulating both active fluids in complex geometries and suspended solutes having complex shape or internal degrees of freedom. Here, we present a novel mesoscopic particle-based algorithm for stochastically simulating active, wet, compressible nematic fluids.

## MATERIALS AND METHODS

Continuum active nematohydrodynamics are described by three transport equations for mass, momentum, and orientational order (*2*). The method introduced here to simulate these equations of motion extends the multiparticle collision dynamics (MPCD) algorithm to active nematohydrodynamics. The active collision operator locally injects energy while conserving momentum. Our purpose is to develop an active MPCD algorithm to reproduce active nematic behavior rather than to present a particular optimization. However, the MPCD algorithm performs favorably with Graphics Processing Unit implementations (*40*, *41*). This section first introduces the conceptual basis of the passive MPCD framework, summarizes the collision operators used, and lastly extends these to an active nematic collision operator.

### Multiparticle collision dynamics

MPCD algorithms discretize continuous hydrodynamic fields into *N* point particles (labeled *i*). Each MPCD particle of mass *m_i_*, position **r**_*i*_, and velocity **v**_*i*_ streams ballistically for a time δ*t* to a new position

$$r_{i}(t+\delta t)=r_{i}(t)+v_{i}(t)\delta t$$ (1)

before undergoing a multiparticle stochastic collision event (*42*).

Although real molecules that constitute a fluid interact with one another via microscopically specific pair potentials, the details of these molecular interactions are commonly inconsequential to the resulting continuum equations of motion in the isotropic hydrodynamic limit. The MPCD method leverages this reality to recover hydrodynamic equations on long time and length scales using an artificial and mesoscopic multiparticle collision operator rather than specific intermolecular potentials between pairs of molecules (*43*). MPCD collision events occur within lattice-based cells (labeled *c* at position **r**_*c*_) defined by a size *a*. In each cell *c*, the instantaneous population *N*_*c*_(*t*)≡*N*(**r**_*c*_;*t*) of particles stochastically exchanges properties through collision operators that respect the relevant conservation laws. Discretizing space into grid cells breaks Galilean invariance. To counter this, a grid shift is applied at each time step (*43*). The collision operator governs the fluid transport coefficients (*43*).

To conserve mass and reproduce the continuity equation $\partial_{t}\rho =-\nabla \cdot (\rho v_{c}^{\text{cm}})$, collision operators must simply leave the number of MPCD particles unchanged between collision events. Similar to other particle-based hydrodynamic solvers, MPCD fluids are not incompressible (*44*).

To conserve momentum, the cell’s net momentum must be unchanged by collisions, which amounts to an unchanged center of mass velocity $v_{c}^{\text{cm}}={\langle v_{i}\rangle }_{c}$ if all the particles have the same mass *m_i_* = *m* (*42*). The average ⟨ · ⟩*_c_* is over the particles within cell *c* at position **r**_*c*_. The average velocity $v_{c}^{\text{cm}}(t)$ is interpreted as the hydrodynamic velocity field at the position of that cell **r**_*c*_. Similarly, constraints to the stochastic exchange of particle velocities lead to conservation of energy and angular momentum and their associated hydrodynamic fields.

In stochastic, particle-based algorithms, diffusion dominates over advection, making these suitable for the small Péclet number limit. This is in contrast to hydrodynamic solvers, such as pseudo-spectral methods (*45*), which do not inherently account for diffusion and, thus, operate in the large Péclet number limit where advection dominates. MPCD allows for both advection and diffusion and, thus, is suitable for simulations of systems with moderate Péclet numbers.

### Angular momentum conserving Andersen MPCD

The continuous velocity field of an active nematic is given by a generalized Navier-Stokes equation, in which the total hydrodynamic stress is the sum of a viscous stress, the orientational elastic stresses of nematic liquid crystals, and an active stress (*2*). To simulate the stresses, MPCD velocities are updated according to

$$v_{i}(t+\delta t)=v_{c}^{\text{cm}}(t)+\Xi_{i,c}$$ (2)

where the collision operator **Ξ**_*i*,*c*_ is a nonphysical exchange of momenta within each cell *c*. The collision operator is designed to be stochastic while constrained to conserve net momentum (and kinetic energy in passive fluids). Through the use of a simple multiparticle collision operator, computationally costly pairwise interactions between fluid molecules can be avoided. Furthermore, the local nature of the collision operators makes the approach amenable to parallelization and improves MPCD’s computational performance (*46*). The Andersen collision operator $\Xi_{i,c}^{0}$ (*47*) is a common example of **Ξ**_*i*,*c*_ for passive, isotropic fluids and has the form

$$\Xi_{i,c}^{0}=\xi_{i}-{\langle \xi_{j}\rangle }_{c}+({\underline{\underline{I}}}_{c}^{-1}\cdot \delta L_{\text{vel}})\times r_{i}^{\prime }$$ (3)

Here, ξ_*i*_ is a random velocity drawn from the Maxwell-Boltzmann distribution for thermal energy *k*_B_ *T*, and ⟨ξ_*j*_⟩_c_ is the cell average. The moment of inertia is ${\underline{\underline{I}}}_{c}=\sum_{j}^{N_{c}}m_{j}({r^{\prime }}_{j}^{2}\underline{\underline{\hat{1}}}-{r_{j}}^{\prime }{r_{j}}^{\prime })$ for point particles in cell *c* relative to their center of mass $r_{c}^{\text{cm}}$, where $r_{i}^{\prime }=r_{i}-r_{c}^{\text{cm}}$.

The third term in the collision operator corrects any spurious angular momentum introduced by the collision operator $\delta L_{\text{vel}}=\sum_{j}^{N_{c}}r_{j}\prime \times (v_{j}-\xi_{j})$.

The conceptual basis of MPCD can be extended from momentum collision operators that reproduce the hydrodynamic velocity field to collision operators for other hydrodynamic fields. In particular, nematic liquid crystals can be simulated via a collision operator to exchange particle orientations (*48*).

### Nematic MPCD

In nematic liquid crystals, the tensor order parameter ${\underline{\underline{Q}}}_{c}$ measures the extent and direction of orientational order through its largest eigenvalue *S_c_* and associated eigenvector ${\underline{n}}_{c}$ in cell *c*. The evolution of ${\underline{\underline{Q}}}_{c}(r_{c};t)={\langle du_{j}u_{j}-\underline{\underline{\hat{1}}}\rangle }_{c}/(d-1)$ can be simulated using passive nematic MPCD by assigning an orientation **u**_*i*_ to each point particle. The identity matrix is denoted $\underline{\underline{\hat{1}}}$. This work focuses on an approach where the orientation is updated through a stochastic nematic multiparticle orientation collision operator (*48*) based on the local equilibrium distribution for the orientation field

$$u_{i}(t+\delta t)=n_{c}(t)+\eta_{i}$$ (4)

The noise **η**_*i*_ is drawn from the Maier-Saupe distribution ∼ exp (β *US*_*c*_[**u**_*i*_·**n**_*c*_]^2^). The width of the distribution about **n**_*c*_(*t*) is controlled by a mean-field interaction constant *U* and inverse thermal energy β = 1/*k*_B*T*_. When β*US_c_* is small, all orientations are equally likely, whereas when it is large, the distribution becomes sharply oriented about **n**_*c*_, governing whether the fluid is in an isotropic or nematic phase. Furthermore, Frank coefficients *K* can be computed as a linear function of β*U* (*48*, *49*).

A two-way coupling is applied between the fluid velocity and orientation. Shear alignment couples the orientation to gradients in the velocity through Jeffery’s equation for a slender rod with tumbling parameter λ (*48*). A relaxation parameter χ tunes the hydrodynamic susceptibility of orientation to velocity gradients. Backflow coupling is applied to the velocity due to the dynamics of the director field. The torques that rotate MPCD particles are balanced by applying an equal and opposite torque to the fluid via a change in linear momentum $\delta L_{\text{ori}}=-\gamma_{R}\sum_{j}^{N_{c}}u_{j}\times {\dot{u}}_{j}$, where γ_R_ is a viscous rotation coefficient, in the collision operator. This contribution is added to the isotropic collision operator $\Xi_{i,c}^{0}$ (Eq. 3) to produce the nematic MPCD collision operator

$$\Xi_{i,c}^{N}=\Xi_{i,c}^{0}+({\underline{\underline{I}}}_{c}^{-1}\cdot \delta L_{\text{ori}})\times r_{i}\prime$$ (5)

Alternative hybrid finite difference/MPCD approaches have been proposed to simulate nematic liquid crystals (*50*, *51*). Nematic MPCD has been used to study nematohydrodynamic fluctuations and correlations (*52*, *53*), electroconvection (*54*), defects around nanocolloids (*49*, *55*), and living liquid crystals (*56*). We now extend the nematic Andersen-thermostatted collision operator to simulate wet active nematics.

### Active nematic MPCD

To make MPCD active, an intrinsically out-of-equilibrium term must be included in the collision operation. We seek an algorithm for momentum conserving (wet) active fluids (*2*); therefore, we propose a collision operator that injects energy but not momentum. The form of the introduced active stress should correspond to a force dipole density that can be extensile or contractile (*2*). As in continuum models of active nematics, this algorithm uses a form for which the local active stress is proportional to the nematic order tensor $∼{\underline{\underline{Q}}}_{c}$ (*57*) or, equivalently, a form for which the force dipole coaligns with the local director **n**_*c*_.

To account for these considerations, the active nematic MPCD (AN-MPCD) collision operator is a linear combination of passive and active contributions

$$\Xi_{i,c}^{A}=\Xi_{i,c}^{N}+\alpha_{c}\delta t(\frac{\kappa_{i}}{m_{i}}-{\langle \frac{\kappa_{j}}{m_{j}}\rangle }_{c})n_{c}$$ (6)

The passive contribution $\Xi_{i,c}^{N}$ is given by Eqs. 3 and 5. The active contribution is composed of two terms: (i) individual impulses (per unit mass) (α_*c*_δ*t*/*m*_*i*_)κ_*i*_**n**_*c*_ on each particle *i*, which represent the active force driving a change in momentum over each time step, and (ii) a term to ensure local conservation of momentum −(α_*c*_δ*t*/〈*m*〉_*c*_)〈κ_*j*_〉_*c*_**n**_*c*_. Thus, as in the Andersen collision operator (Eq. 3), any residual impulse is removed. The activity term (α_*c*_δ*t*/*m*_*i*_)κ_*i*_**n**_*c*_ is composed of three factors: (i) α_*c*_, (ii) κ_*i*_**n**_*c*_ = ±**n**_*c*_, and (iii) δ*t*/*m*_*i*_.

i) The factor α_*c*_(*t*) ≡ α(**r**_*c*_;*t*) represents the local active dipole strength in cell *c*. The cellular activity α*_c_* is found by summing over the individual activity value α*_j_* of every MPCD particle *j* within each cell

$$\alpha_{c}=\sum_{j=0}^{N_{c}}\alpha_{j}$$ (7)

**Fig. 1.**
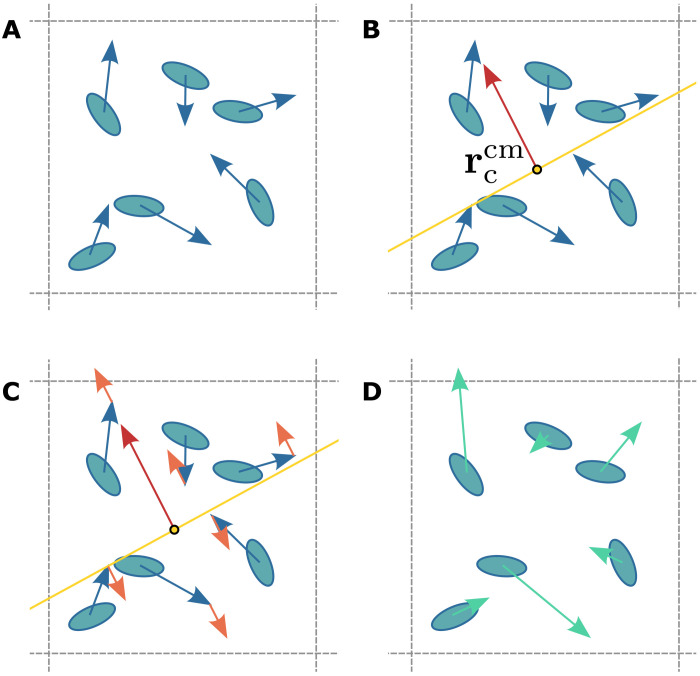
Schematic representation of the active portion of the collision operator (Eq. 6). (**A**) Particles are binned into cells, with index *c* at position **r**_*c*_. Each particle has an internal orientation **u**_*i*_ schematically shown as an ellipsoidal shape and velocity **v**_*i*_ denoted by a blue arrow. (**B**) The local nematic director **n**_*c*_ (red arrow) and center of mass $r_{c}^{\text{cm}}$ of the cell define a plane with approximately half the particles above and half below (yellow plane). (**C**) Each particle is subject to an impulse away from the plane (orange arrows). (**D**) Particles stream with their new velocities (green arrows).

This represents a local activity that is directly proportional to the local density of active agents within a given MPCD cell. In this model, the presence of more active agents produces higher activity (*58*). Positive particle activity α_*j*_ corresponds to extensile active nematics, whereas negative values correspond to contractile activity. Here, all particles have the same activity α_*j*_ = α.

ii) The factor $\kappa_{i}(r_{i},r_{c}^{\text{cm}},n_{c})n_{c}$ gives the direction of the active force acting on particle *i*. In an active nematic, the activity is dipolar and parallel to the local nematic director **n**_*c*_(*t*). Whether the impulse on each individual particle is parallel or antiparallel to the cell director is set through $\kappa_{i}(r_{i},r_{c}^{\text{cm}},n_{c})=\pm 1$. This parallel/antiparallel coefficient evenly splits the particles within cell *c* into those that are driven “forward” (κ*_i_* = +1) and those that are kicked “backward” (κ*_i_* = −1). To do this, the collision operator considers the plane passing through the center of mass $r_{c}^{\text{cm}}$ with surface normal **n**_*c*_. Generally, half of the MPCD particles within cell *c* are on either side of this plane. The parallel/antiparallel coefficient κ*_i_* = +1 for particles above the plane and κ*_i_* = −1 for particles below the plane. This algorithm is visually illustrated in Fig. 1.

iii) The factors of δ*t* and $m_{i}^{-1}$ ensure that α*_c_* has units of force.

Including the active collision operator (Eq. 6) in the nematic MPCD algorithm results in a wet, particle-based, mesoscale, active nematic simulation method applicable to moderate Péclet numbers.

### Simulation units and parameters

Distances are reported in units of MPCD cell size *a* ≡ 1, and energy is reported in units of thermal energy *k*_B_*T* ≡ 1. This work considers only a single species, such that all particles have the same mass *m_i_* = *m* ≡ 1 and activity α*_i_* = α ∀*i*. Thus, the local activity in cell *c* is proportional to the local density α*_c_* = α*N_c_*, meaning that activity varies with any variation in density. Activity is given in MPCD units of force *ma*/τ^2^ = *k*_B_*T*/*a* ≡ 1. The MPCD time scale is thus $\tau =a\sqrt{m/k_{B}T}\equiv 1$. Simulation parameters are expressed in these MPCD simulation units. Simulations are run in two dimensions in systems of size ℓ_sys_ × ℓ_sys_ with periodic boundary conditions and ℓ_sys_ = 200, except when explicitly stated otherwise. Cartesian axes are denoted $\hat{x}$ and $\hat{y}$. The system-averaged fluid density is represented by the average particle density per cell, ⟨*N_c_*⟩ = 20. The time step is set to δ*t* = 0.01, and, unless otherwise stated, simulations use a warm-up phase of 10^5^ time steps and a data production phase of 5 × 10^5^ time steps.

MPCD particles are initialized randomly within the control volume with random velocities, and orientations are initialized uniformly along the $\hat{y}$ axis. The simulations are performed in the nematic phase by setting *U* = 10, with a rotational friction γ_R_ = 0.01 and hydrodynamic susceptibility χ = 0.5. The tumbling parameters λ = 2 and 1/2 are simulated as representative of shear-aligning and flow-tumbling regimes, respectively. The shear-aligning regime (λ = 2) is assumed, except where explicitly stated.

The activity ∣α∣ < 1 is varied across four orders of magnitude to identify behavioral regimes of the algorithm. The activity is extensile (α > 0), unless otherwise stated. There exists a minimum activity α_eq_ below which the Andersen thermostat can absorb the active energy injection, yielding effective equilibrium behavior. Active turbulence, with its associated steady-state population of defects, arises at some greater activity α_turb_ ≥ α_eq_ and exists for a finite activity regime α_turb_ ≤ α ≤ α_†_, where α_†_ denotes the end of the hydrodynamic active turbulence scaling regime.

## RESULTS

We now present the results of AN-MPCD simulations by first demonstrating that a hydrodynamic instability causes a proliferation of topological defects. The dependence of the emergent flows on the activity α is systematically explored by considering the structure of the flow fields. The results show that AN-MPCD has an operational active turbulence–scaling regime, in which fully developed active turbulence exists with a characteristic emergent length scale ℓ_α_ that scales with activity. The effects of activity on the local density are characterized, and it is found that AN-MPCD exhibits giant number fluctuations at sufficiently high activity.

### Hydrodynamic instability and defect unbinding

Experiments (*59*) and continuum simulations (*60*) of active nematic systems exhibit activity-induced instabilities, which generate inhomogeneities in the nematic order. This leads to a steady-state population of topological defects and active turbulence (*22*). The AN-MPCD algorithm reproduces this two-stage development of active turbulence through a hydrodynamic instability and defect pair creation.

**Fig. 2.**
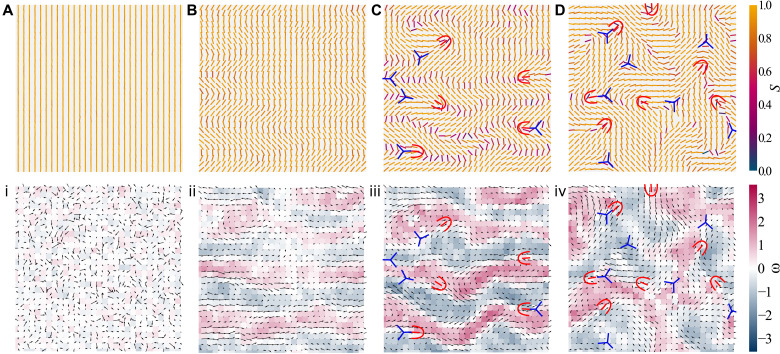
Instabilities lead to defect pair creation and active turbulence. (**A** to **D**) Snapshots of the local director field **n**_*c*_(**r**_*c*_;*t*) colored by scalar order parameter *S*_*c*_(**r**_*c*_;*t*). (**i** to **iv**) Snapshots of vorticity ω_*c*_(**r**_*c*_;*t*) with arrows showing velocity **v**_*c*_(**r**_*c*_;*t*), simulated in a system size of ℓ_sys_ = 30 and extensile activity α = 0.03 from movies S1 and S2. Each line segment, cell, and arrow corresponds to one MPCD cell, and no smoothing has been applied to the images. (A, i) Time step 0: The director is initialized in a fully aligned configuration, and thermal noise perturbs the director field. (B, ii) Time step 110 δ*t*: Extensile bend instability causes high-bend kink walls to form perpendicular to the global director with net force density parallel to the bend. (C, iii) Time step 160 δ*t*: Defect pairs unbind within walls. +1/2 and −1/2 defects (red and blue, respectively) are highlighted to guide the eye. (D, iv) Time step 245 δ*t*: Steady-state defect population arises, leading to active turbulence.

This process is depicted by simulation snapshots in Fig. 2 from movie S1. The simulation is initialized in a fully ordered state with all nematogen orientations parallel to the $\hat{y}$ axis, i.e., $u_{i}=\pm \hat{y}$ ∀ *i* (Fig. 2A, i), and the warm-up period is eschewed. At early times, the hydrodynamic instability manifests as kink walls—narrow, initially parallel, well-spaced, alternating regions of high-bend deformation (Fig. 2B). In Fig. 2B (ii), this is seen as bands of alternating vorticity. For extensile activity, fluctuations in bend produce active forces proportional to $∼\alpha \nabla \cdot \underline{\underline{Q}}$, which drive spontaneous flows along the kink walls (Fig. 2B, ii). However, these self-driven streams exacerbate the inhomogeneities through the backflow, which, in turn, increases the active forces causing the instability (*61*, *62*). For sufficiently high activity, this hydrodynamic bend instability is responsible for the formation of topological defect pairs (Fig. 2C, iii). Beyond a certain point, the total distortion free energy cost along a kink wall is higher than the cost of two oppositely charged defects, and a defect pair is spontaneously created (*22*). The defect core size is comparable to the MPCD cell size *a*. Although there is an elastic restoring force, the +1/2 defect is a self-propelled quasiparticle, and it moves along the kink wall, becoming unbound from the non-motile −1/2 defect (Fig. 2D, iv) (*22*, *63*).

**Fig. 3.**
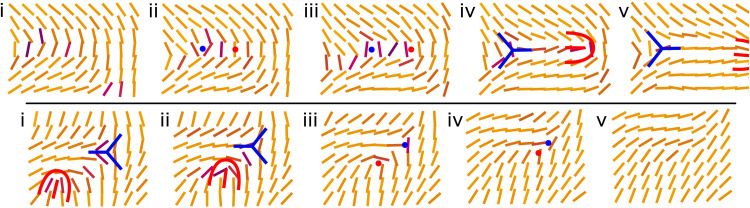
AN-MPCD exhibits pair creation and annihilation events. Snapshots of the local director field **n**_*c*_(**r**_*c*_;*t*) depicting creation (top row) and annihilation (bottom row) events in extensile AN-MPCD. Snapshots are taken from the same simulation as shown in Fig. 2 and movie S1. Each line segment corresponds to the director in one MPCD cell. The frame rate between snapshots is 5δ*t*. Positive +1/2 (red) and negative −1/2 (blue) defects are highlighted to guide the eye. Top row: (**i**) An initial kink wall. (**ii** and **iii**) The bend instability results in net force parallel to the bend, exacerbating the deformation. (**iv**) Spontaneous creation of a ±1/2 defect pair. (**v**) The +1/2 defect is self-motile and departs from the creation event site. Bottom row: (**i**) Proximate +1/2 and −1/2 defects. (**ii**) The self-motile +1/2 defect approaches the −1/2 defect. (**iii**) As the defects approach, they begin to lose their distinct shapes. (**iv**) Defect annihilation reduces nematic distortion. (**v**) Locally ordered nematic remains after an annihilation event.

A pair creation event is illustrated in detail in the top row of Fig. 3. Along a kink wall, a pair of ±1/2 defects is formed, conserving the net topological charge. The motility of +1/2 defects causes the pair to unbind. As the motile +1/2 defect travels along the kink wall, it alleviates the deformation free energy, leaving the region of uniform order in its trail (Fig. 3, top row) but generating local vorticity, which, in turn, perturbs nearby kink walls, disordering the orientation of future defect pair unbinding events giving rise to active turbulence (see Fig. 2, C and D, ii to iv). Although the activity generates defects, they come with an inherent free energy cost due to the deformation they induce in the director field, and, as such, they annihilate to minimize the deformation cost (Fig. 3, bottom row). As a +1/2 defect approaches a −1/2 defect, they annihilate and order the local region. Continual creation and annihilation leads to a steady-state defect population.

### Steady-state defect population

To assess the degree of nematic ordering within the steady-state AN-MPCD fluid, the spatial autocorrelation of the director field *C*_nn_(*R*) as a function of radial separation *R* is considered (eq. S4). Since the director is apolar, **n** → −**n**, the decorrelation limit corresponds to *C*_nn_(*R*) → 2/3. For the lowest activities considered (α≲α_eq_ = 10^−3^), the correlation remains high even as *R* → ℓ_sys_ (fig. S1A). In these cases, activities less than α_eq_ are negligible, and the injected energy can be absorbed by the thermostat so that the system remains globally ordered. This is seen in movie S3, where the nematic director field remains oriented primarily in the $\hat{y}$ axis, with slight thermal fluctuations. For the low-activity regime (α_eq_≲α≲α_turb_, where α_turb_≃10^−2^), the director field decorrelates somewhat but does maintain long-range correlations/anti-correlations, which represent the persistent existence of kink walls with a characteristic separation length (movie S4). Only once α ≳ α_turb_ do the correlation functions fully decorrelate to lim_*R*→∞_*C*_nn_(*R*)→ 2/3 and dip below the long-range value, as observed in continuum simulations of fully formed active turbulence (movies S5 and S6) (*24*). The activity α_turb_ = 10^−2^ represents the lower threshold between fully developed active turbulence and kink walls for this system size. In the active turbulence regime, α_turb_≲α, we find that the correlations belong to the same class of functions by rescaling the separation by the nematic decorrelation length (fig. S1B). Rescaling collapses the curves for α ≳ α_turb_.

**Fig. 4.**
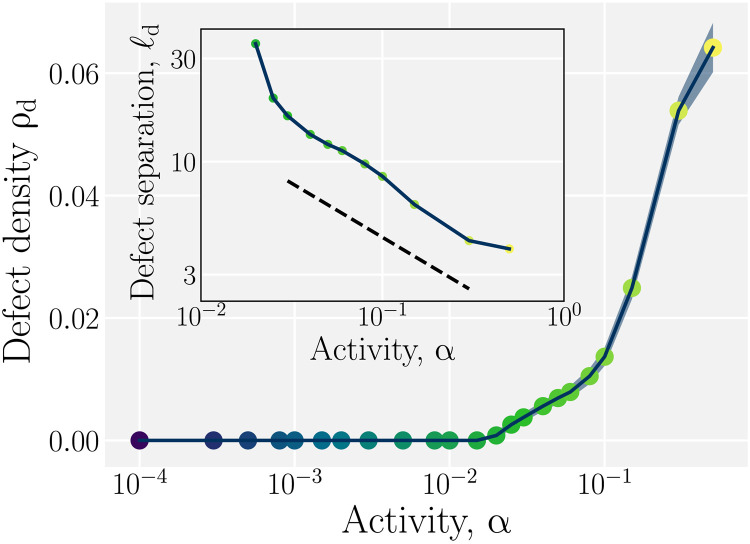
Defect population increases and separation decreases with activity. Steady-state density ρ_d_ of +1/2 defects as a function of activity. Error bars denote the standard deviation of the number of defects. Inset: Root mean square separation between all defects $\ell_{d}=\rho_{d}^{-1/2}$. The dashed line indicates a scaling of ℓ_d_ ∼ α^μ^ with μ = −1/2.

Consistent with the measured correlation functions, the number of defects is found to be nonzero only for sufficiently strong activity α ≳ α_turb_, where the minimum activity to observe turbulence is α_turb_ = 2.5 × 10^−2^ for ℓ_sys_ = 200 (Fig. 4). Above α_turb_, the mean defect density ρ_d_ rises rapidly, and the standard deviation (SD) increases discontinuously from zero. The root mean square separation between ±1/2 defects, $\ell_{d}=\rho_{d}^{-1/2}$, decreases accordingly (Fig. 4, inset). The scaling ℓ_d_ ∼ α^μ^ with μ = − 1/2 is expected from dimensional analysis for a range above α_turb_ (eq. S1), and the simulation results are consistent with the expected scaling within the fully formed turbulence region α_turb_ ≤ α ≤ α_†_ (Fig. 4, inset). Thus, continual creation and annihilation leads to a steady-state defect population in AN-MPCD characterized by the competition between activity and nematic elasticity (eq. S1).

**Fig. 5.**
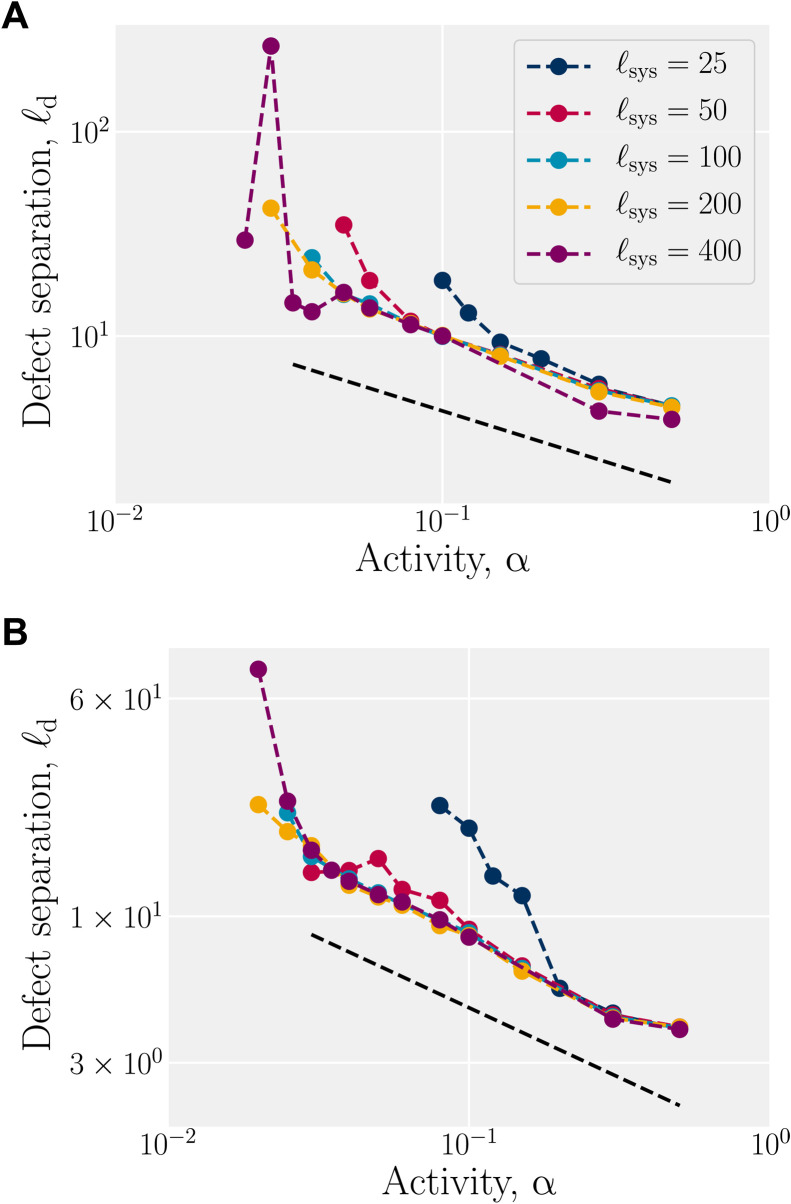
System size has little effect on defect separation. (**A**) Steady-state mean separation of defects $\ell_{d}=\rho_{d}^{-1/2}$ in the tumbling regime (λ = 1/2) as a function of activity for various system sizes ℓ_sys_. (**B**) Same as (A) for shear-aligning active nematics (λ = 2). The dashed lines indicate a scaling of ℓ_d_ ∼ α^μ^ with μ = −1/2.

To assess system size effects, larger and smaller systems ℓ_sys_ ∈ {400,200,100,50,25} for both tumbling (λ = 1/2 in Fig. 5A) and shear-aligning (λ = 2 in Fig. 5B) behaviors are simulated. In both cases, the system size shifts the activity at which defect pairs unbind and the range of activities for which the active turbulence scaling law holds is extended for larger system sizes.

### Spontaneous flow

The previous section demonstrates that the AN-MPCD algorithm reproduces the hydrodynamic instability, topological defect propagation, the self-motility of +1/2 defects, and the expected scaling of the characteristic length scale as measured by the mean separation between defects. This section explores the spontaneous flows and confirms that AN-MPCD reproduces the properties of fully developed active turbulence across a range of activities above α_turb_.

**Fig. 6.**
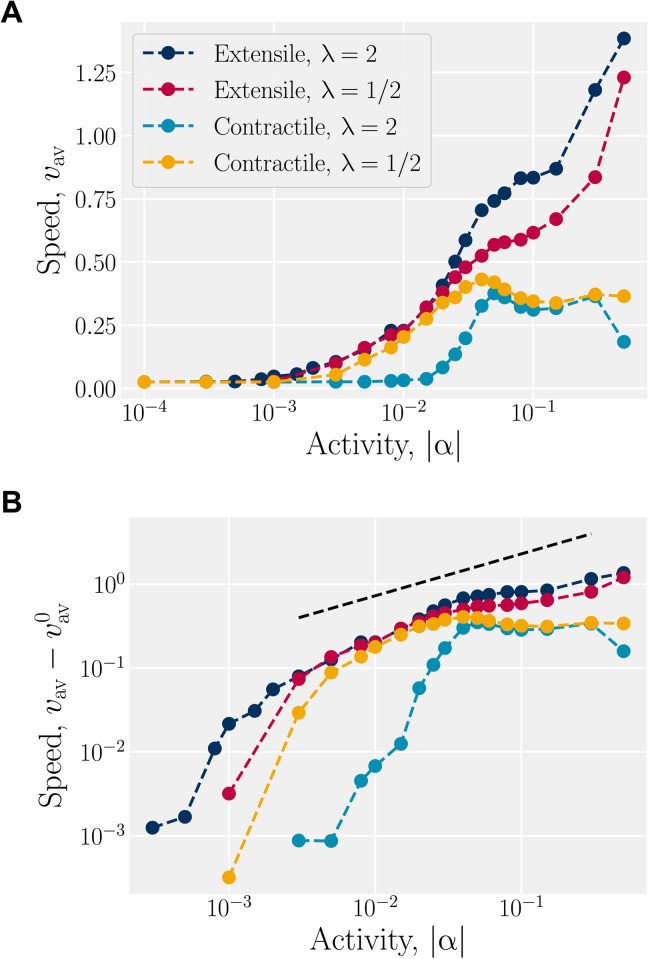
Activity generates a spontaneous flow. (**A**) Root mean square velocity for extensile and contractile active nematics in both the flow-tumbling (λ = 1/2) and shear-aligning (λ = 2) regimes. (**B**) Root mean square velocity on a log-log scale, with the ambient thermal speed subtracted. The dashed line represents a scaling of *v*_av_ ∼ α^γ^ with γ = 1/2.

The magnitude of the active flows is quantified by measuring the steady-state, spatiotemporally averaged, root mean square velocity *v*_av_ (Fig. 6A). Since AN-MPCD is a thermalized method, lim_α→0_*v*_av_ ≠ 0 and a small but nonzero value of *v*_av_ is measured below α_eq_ = 10^−3^ (Fig. 6A and movie S7). Above α_eq_, the average speed rises rapidly—more rapidly than predicted by eq. S2 (Fig. 6B). This is consistent with fig. S1: Between α_eq_ and α_turb_, the activity is sufficient to generate kink walls (movie S4) and spontaneous flow (movie S8) but insufficient to produce defects and active turbulence. Thus, the scale of the characteristic speed rises faster than when active turbulence is fully formed. Active turbulence is fully formed at α_turb_≃10^−2^ (movies S9 and S10), and the scaling *v*_av_ ∼ α^γ^ is seen to be in agreement with the ideally expected value of γ = 1/2 from dimensional analysis (eq. S2), shown as a dashed line in Fig. 6B, for the region α ≳ α_turb_. Fitting the exponent gives γ = 0.45 ± 0.05 in the region α ∈ [0.04,0.15].

**Fig. 7.**
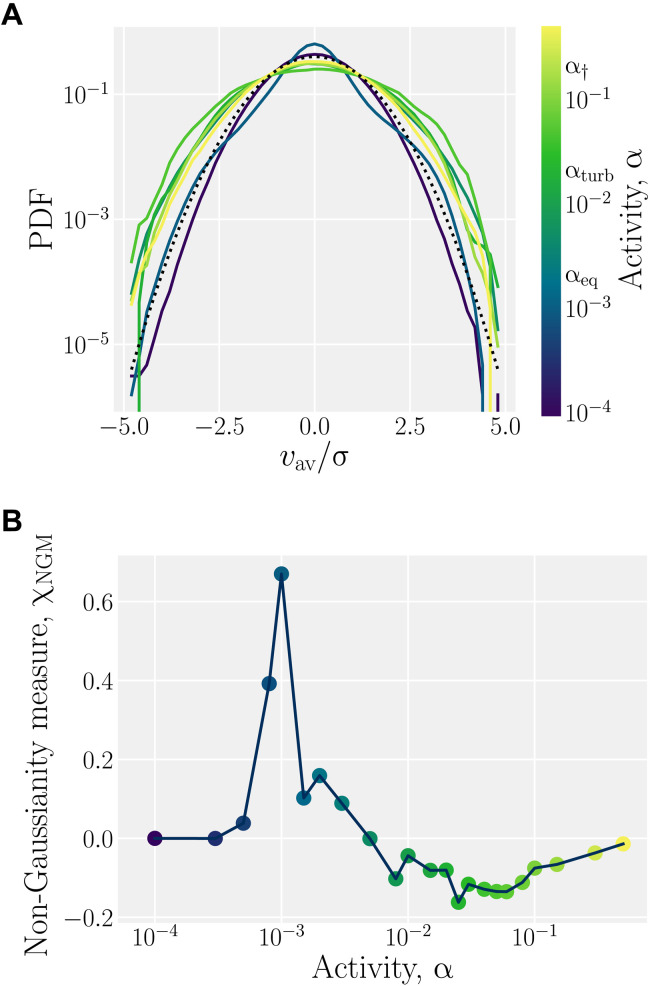
Activity broadens velocity distributions in AN-MPCD. (**A**) Probability density function (PDF) of the local average of velocity components, rescaled by the standard deviation s, for λ = 2 and ℓ_sys_ = 200. The black dotted line is a standard Gaussian distribution. (**B**) Non-Gaussianity measure χ_NGM_ of the distributions in (A).

As activity increases, *v*_av_ does too, representing the broadening of the distribution of velocities. However, this is not the only effect; the probability density function also changes shape (Fig. 7A). For the lowest activities (α ≲ α_eq_), the distributions of the $\hat{x}$ and $\hat{y}$components are Gaussian. For higher activities (α ≳ α_eq_), the distribution exhibits longer tails, and the kurtosis is larger than for normal distributions, which is indicative of the out-of-equilibrium behavior and correlated flow fields. To quantify the degree of non-equilibrium behavior, a non-Gaussianity measure (χ_NGM_ given by eq. S6) for the velocity distributions is considered in Fig. 7B. For the lowest activities, χ_NGM_ = 0, corresponding to a true Gaussian distribution for velocity; however, this quickly peaks around α ≃ α_eq_. Note that our non-Gaussianity measure is directly related to the Binder cumulant (*64*), and the peak is indicative of a discontinuous onset of activity-induced flows. The positive peak in χ_NGM_ corresponds to the single narrower distribution in Fig. 7A. As activity increases past α_eq_, the non-Gaussianity measure drops to negative values and remains negative throughout the turbulence scaling region α_turb_ ≲ α ≲ α_†_, corresponding to the broadening velocity distributions in Fig. 7A. Last, for α_†_ ≲ α, the non-Gaussianity measure increases toward χ_NGM_ → 0, indicating the breakdown of the algorithm.

To quantify the flow structures responsible for the non-Gaussianity, the velocity autocorrelation functions *C_vv_*(*R*) are measured (fig. S2A). Correlation functions in which thermal, rather than hydrodynamic, effects dominate are neglected. By fitting an exponentially decaying function, the characteristic velocity lengths are obtained (fig. S2B). For α ≲ α_eq_, activity is insufficient to induce hydrodynamic effects. Just as in Fig. 6, for α_eq_ ≲ α ≲ α_turb_, hydrodynamic effects are apparent, in that there is a characteristic hydrodynamic length scale. However, there is still insufficient activity to drive active turbulence at these low activities; thus, the decorrelation length does not yet scale with activity. Only once α ≳ α_turb_ does the velocity decorrelation length scale decrease with activity. In this regime, the decrease in length scale is seen to correspond to the ideal expectations ℓ*_v_* ∼ α^μ^ where μ = −1/2 from dimensional analysis (eq. S1), shown as a dashed line, and fit to μ = −0.48 ± 0.05 in range [0.025,0.3]. Compared to Fig. 4, the director and velocity decorrelation length scale both scale as expected within the fully formed active turbulence regime, α ≳ α_turb_. Correspondingly, the measured length scale collapses the velocity-velocity correlation lengths in the fully formed active turbulence regime of α ≳ α_turb_ (fig. S2C).

**Fig. 8.**
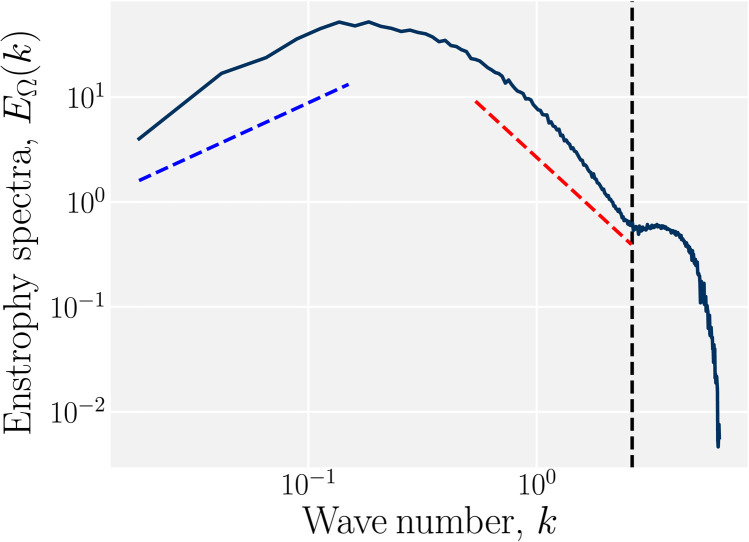
Enstrophy spectra for activity α *=* 0.08 in a system of size ℓ_sys_ *=* 400. Scalings of ∼*k*^1^ at low wave numbers (blue dashed line) and ∼*k*^−2^ at intermediate wave numbers (red dashed line) guide the eye. At the highest wave numbers, there exists a secondary structure due to the MPCD cell size *a*, which appears because vorticity is computed on the MPCD lattice. The wave number at which vorticity is computed is marked as the vertical black dashed line.

Active nematics do not have the scale invariance that inertial turbulence does (*23*, *25*). This is because energy is dissipated at the scale it is injected in, impeding energy cascades. In particular, the length scale from eq. S1 represents the characteristic vortex size, and so considering the amount of enstrophy as a function of wave number (eq. S5) reveals the spatial structure of active turbulence. At small wave numbers, the enstrophy rises linearly with wave number as ∼*k*^+1^ (Fig. 8). The computed scaling at low wave numbers is 0.84 ± 0.13 in the range [0.02,0.16]. On the other hand, at intermediate wave numbers, the enstrophy decreases as ∼*k*^−2^ (Fig. 8), which is numerically found to be −2.01 ± 0.03 in the range [0.54,2.6], consistent with expectations. This change of sign in the scaling of the enstrophy spectra is expected to occur at *k* > *k*_α_, the wave number representing the characteristic vortex size (*25*). The vortex structure results in a non-monotonic enstrophy spectra with a maximum corresponding to the characteristic vortex diameter. At the largest wave numbers, the signs of the MPCD cell discretization size *a* are apparent as a secondary peak for *k* ≥ *k_a_* ≈ 2π/3*a* (Fig. 8). This is because nearest-neighbor MPCD cells are used to calculate the local vorticity.

These results demonstrate that the AN-MPCD algorithm generates spontaneous flows for sufficiently large activities. The scale of these flows increases rapidly at first, when the ratio of system size and activity are insufficient for fully developed turbulence, but for sufficient activity, the characteristic velocity grows according to the expected scaling. The autocorrelation functions and enstrophy spectra demonstrate that AN-MPCD reproduces the essential properties of fully developed active turbulence with flow structures that statistically match expectations. However, AN-MPCD is a point particle–based mesoscale method and, as such, also has properties expected of active particle models.

### Density and activity fluctuations

**Fig. 9.**
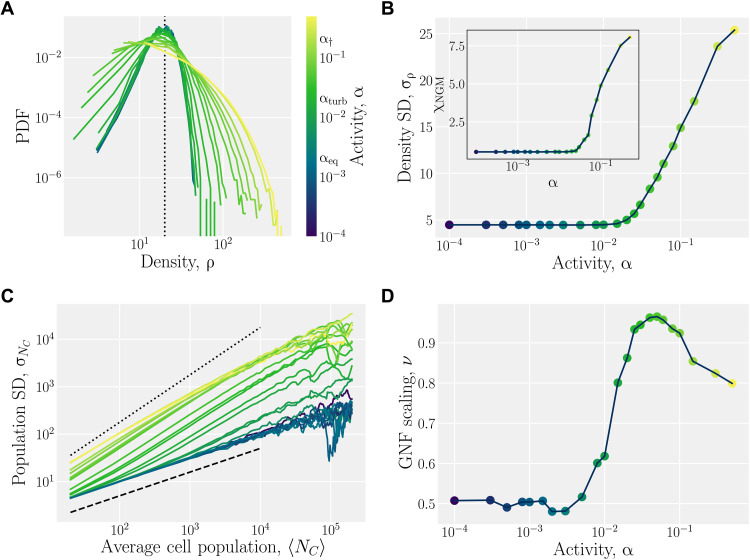
AN-MPCD exhibits giant number fluctuations. (**A**) Probability density function (PDF) of the cell density. The average cell density ⟨*N_c_*⟩ = 20 is shown as a dotted black line. Local activity is directly proportional to density. (**B**) Standard deviation (SD; σ_ρ_) of the cell density as a function of activity. Inset: Non-Gaussianity measure χ_NGM_ of cell density PDF (A). (**C**) SD of population shown as a function of the average cell population. Scalings of σ*_N_c__* ∼ 〈*N_c_*〉*^ν^* with ν = 1/2 (black dashed line) and ν = 1 (black dotted line). (**D**) The scaling exponent ν of (C) measured at the low cell population limit.

As a mesoscale algorithm between the continuum and microscopic limits, AN-MPCD exhibits both the active turbulence predicted by continuum approaches and the density fluctuations of particle models. Since MPCD is composed of point particles and obeys an ideal gas equation of state (*43*, *44*), it is a compressible fluid, as illustrated by the probability distribution of the density (Fig. 9A). The density fluctuations directly lead to activity fluctuations, since α*_c_* = α*N_c_* (Eq. 7 for constant particle activity).

In the low-activity limit α ≪ α_eq_, the fluid has a relatively broad distribution of particles with a mean ρ = ⟨*N_c_*⟩/*a*^2^ = 20 and a standard deviation σ*_N_c__* ≃ 4.5 (Fig. 9, A and B). At the lowest activities, the mean coincides with the mode at *N_c_* = 20, and the distribution is relatively Gaussian, with a slight positive skew. At these low activities, the distribution does not change as a function of activity; however, once α ≥ α_turb_, the standard deviation begins to rise (Fig. 9B).

**Fig. 10.**
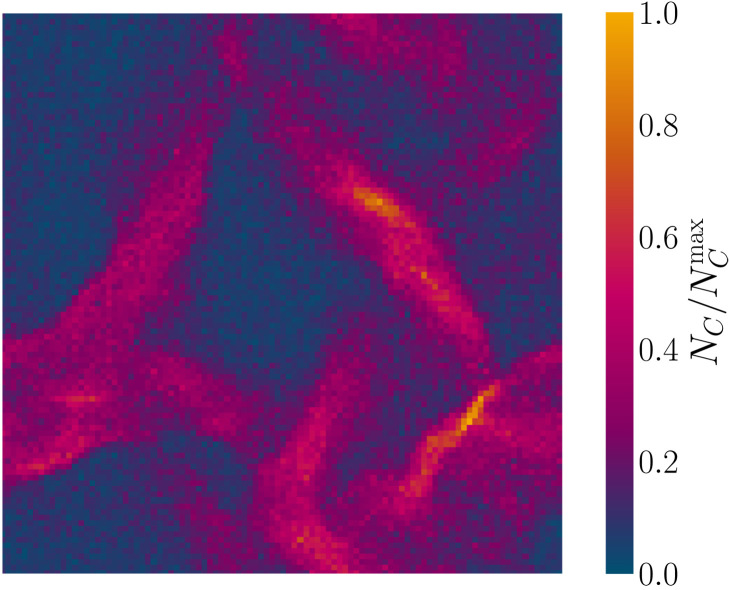
AN-MPCD exhibits high-density bands within a dilute nematic gas. Snapshot of the cell density field of an AN-MPCD system (ℓ_sys_ = 100), α = 0.08, and average cell density ⟨*N_c_*⟩ = 20 from movie S11. $N_{C}^{\text{max}}$ is the instantaneous maximum cell population.

The distribution not only broadens but also changes shape with higher moments of the distribution growing more rapidly (Fig. 9A). Once the standard deviation begins to grow, the non-Gaussianity parameter χ_NGM_ also increases with activity (Fig. 9B, inset). This represents the widening tails of the density distributions in Fig. 9A. At high activities, the likelihood of finding regions with either much higher or much lower density than the mean is increased. This can be seen at high activities α ≳ α_†_ with not only empty cells but also high-density bands within a sparse nematic gas (Fig. 10 and movie S11). Since activity is proportional to density via Eq. 7, these are also bands of high activity. As the activity is raised further, corresponding with broader density distributions, these bands appear narrower as a consequence (movie S12). The bands exhibit spatiotemporal chaos through elongation, splitting and merging, reminiscent of particle-based dry active nematic models (*17*, *65*). However, the AN-MPCD algorithm is not observed to exhibit as substantial a decrease in nematic ordering in the gas-like phase (fig. S3).

To better understand the variation of MPCD particles, consider the number fluctuations within subdomains. From eq. S7, the fluctuations scale with the mean as σ*_N_c__* ∼ ⟨*N_c_*⟩*^ν^* with ν = 1/2 in thermal equilibrium. At low activities (α ≲ α_turb_), the fluctuations obey the central limit theorem with ν = 1/2 (Fig. 9, C and D). However, for activities in the fully developed turbulence regime, density fluctuations increase and cross over to anomalous behavior. As ν → 1, giant number fluctuations dominate the statistics, indicating highly out-of-equilibrium behavior within the active turbulence scaling regime α_turb_ ≲ α ≲ α_†_ (Fig. 9D). The eminence of non-equilibrium particle number fluctuations in AN-MPCD highlights the mesoscale nature of the algorithm. AN-MPCD has properties of both active particle ensembles and active fluids.

The exponent ν has a maximum at α = α_†_ ≃ 3 × 10^−1^. The decrease in ν past this point can be explained by considering how the average nematic order parameter *S* varies with activity (fig. S3). For the near-equilibrium regime of α ≲ α_eq_, the nematic order in the system is at its highest and is effectively constant. The system then transitions to fully formed active turbulence regime α_eq_ ≲ α ≲ α_†_, and the nematic order transitions to another plateau at α ≃ α_turb_. At the highest activities, the nematic order drops off sharply for α_†_ ≲ α, corresponding to the decrease in giant number fluctuations, ν (Fig. 9D and movies S13 to S16). High activity partially disorders the orientation partially by causing local regions of low density, which are effectively below the isotropic-nematic transition point, leading to a drop in the nematic order. Conversely, the drop in nematic order causes the active nematic dipole to be more broadly distributed, which more randomly disperses MPCD particles, leading to less clustering and a slightly more homogeneous density structure.

It is thus natural to consider whether the density fluctuations of AN-MPCD affect macroscopic flow observables, and so we consider cross-correlations with the density. However, neither the scalar order parameter *S* nor the speed is found to covary with the density. The order *S* and density *N_c_* exhibit covariances that are never larger than a single percentile for all activities, indicating that the two fields are independent. This is similarly true for the covariance of the speed with density. Only the autocorrelation of the density shows substantial variation (fig. S4). For α ≲ α_turb_, the density-density autocorrelation *C_N_c_N_c__*(*R*) ≃ 1 even in the far field, indicating that the density is homogeneous throughout space (fig. S4). However, once α ≳ α_turb_, it begins to decorrelate. As activity increases, this decorrelation value becomes smaller and smaller, indicative of the degree of density fluctuation in the system, and the rate of decorrelation increases with activity, reflecting the characteristic length scale of the high-density bands. This indicates that there is no meaningful correlation between the density and the flow or nematic order, suggesting that, while density fluctuations are large, they do not significantly affect the active nematic fields.

## DISCUSSION

We have proposed and quantified a mesoscopic, particle-based algorithm for simulating wet active nematics. It is an extension of the nematic version of the MPCD (N-MPCD) method for moderate Péclet numbers that accounts for active force dipoles. This active-dipole contribution to the MPCD collision operator injects kinetic energy but conserves translational momentum. The active dipole is aligned with the local nematic director. The strength of the force dipole is computed from the particles within each cell, resulting in density-dependent local dipole strength.

This active nematic momentum collision operator generates spontaneous flows and hydrodynamic instabilities, leading to defect unbinding and active turbulence. Activity is found to be negligible below α_eq_, because the Andersen thermostat absorbs the injected energy. In this negligible-activity limit, the fluid is indistinguishable from a passive nematic. For weak activity (α_eq_ ≤ α ≤ α_turb_), spontaneous flows arise, but the characteristic length scale of the activity is comparable to the system size, and so active turbulence is not fully developed. In this regime, defect pair unbinding is rare, and flow correlations span the entire system. Only as the activity approaches α_turb_ does the fluid begin to exhibit the aspects of fully developed active turbulence. In the active turbulence regime, the characteristic length scale, as measured from the defect separation, scales with activity as predicted by dimensional analysis. Likewise, the magnitude of the fluid velocity scales with activity with an exponent comparable to the ideal dimensional analysis prediction.

In addition to exhibiting the traits of active turbulence as expected for a continuum model of active fluids, AN-MPCD also has characteristics of active particle models. Most prominently, the local density of MPCD particles has begun to exhibit giant number fluctuations. The concurrence of active nematic fluid properties and active particle properties highlights the mesoscale nature of AN-MPCD—MPCD is a coarse-grained algorithm that spans the intermediate scales between the microscopic and hydrodynamic limits.

Much work has been accomplished by studying the fundamental properties of active fluids (*2*, *3*). However, soft condensed matter physics is often not principally interested in the dynamics of a background solvent itself. Rather, relaxation dynamics, self-assembly, transport of complex solutes, or other multiscale phenomena are the subjects of physical interest. Passive versions of the MPCD algorithm have been widely used to simulate suspensions within a background fluid, including polymers (*39*, *66*, *67*), polyelectrolytes (*68*), catalytic surfaces (*69*), colloids (*49*, *55*, *70*), and swimmers (*71*–*73*). AN-MPCD opens the door to simulating such complex particles embedded within a spontaneously flowing active medium. Future work could simulate Janus particles (*32*) or other anisotropic passive colloids, including flexible filaments or even polymeric materials (*39*). Similarly, studies of driven particles within active nematics (*33*, *34*) could be extended. AN-MPCD could be used to study suspensions of active particles, such as swimming bacteria, suspended in an active solvent with a different activity or symmetry—e.g., pushers in contractile nematics. By bridging microscopic models, such as active Brownian particles, and macroscopic models, such as the Toner-Tu equation, AN-MPCD offers a pathway for numerical studies of novel active hybrid materials.

## Supplementary Materials

### This PDF file includes:

Analysis Methods Figs. S1 to S4 References
