# Supplementary material for: Mesoscopic simulations of active nematics
Source: Sci Adv. 2022 Aug 24;8(34):eabo5788. doi: 10.1126/sciadv.abo5788 (PMC9401632; doi:10.1126/sciadv.abo5788)
Supplement: Supplementary file 1 — Analysis Methods Figs. S1 to S4 References [file sciadv.abo5788_sm.pdf]

Science Advances
AAAS

Supplementary Materials for

Mesoscopic simulations of active nematics

Timofey Kozhukhov and Tyler N. Shendruk

Corresponding author: Tyler N. Shendruk, t.shendruk@ed.ac.uk

Sci. Adv. **8**, eabo5788 (2022)
DOI: 10.1126/sciadv.abo5788

The PDF file includes:

Analysis Methods
Legends for movies S1 to S16
Figs. S1 to S4
References

Other Supplementary Material for this manuscript includes the following:

Movies S1 to S16

I. ANALYSIS METHODS

I.1. Characteristic Activity Scales

Active nematic MPCD simulations are expected to possess a number of length scales. The algorithm itself has two; the system size ℓ_{sys} and cell size $a \equiv 1$. Additionally, an incompressible active nematic fluid has two: (i) the passive nematic persistence length which is proportional to the defect core size in the nematic phase; and (ii) the active length scale in fully developed mesoscale turbulence, which arises when the nematic elastic stress balances the active stress

$$\ell_\alpha \sim \sqrt{\frac{K}{\alpha}}, \tag{SI 1}$$

where K is the Frank elastic coefficient, which is directly proportional to the mean-field potential U in the nematic MPCD algorithm. From this dimensional analysis, the idealised active length scales as $\ell_\alpha \sim \alpha^\mu$ with the expected power law $\mu = -1/2$ in the regime of fully developed active turbulence. However, it has been demonstrated that μ can saturate if the activity is not sufficiently large and the nematic structure spans a substantial fraction of the system size [74].

Dimensional analysis also reveals an ideally expected characteristic active velocity scale

$$v_\alpha \sim \frac{|\alpha|\,\ell_\alpha}{\eta}, \tag{SI 2}$$

which arises when active stress is balanced by the viscous stress, characterised by the viscosity η. This is comparable to the speed of self-propelled $+1/2$ defects [21]. Since Eq. SI 2 includes both a direct factor of activity and an indirect factor through the active length scale (Eq. SI 1), the velocity scales as $v_\alpha \sim \alpha^\gamma$, where ideally $\gamma = 1/2$. A mesoscale algorithm for simulating the hydrodynamic limit of active nematics should be consistent with $\mu \approx -1/2$ and $\gamma \approx 1/2$ in the regime of simulating fully developed turbulence (*i.e.* $\alpha_{\text{turb}} \leq \alpha \leq \alpha_\dagger$).

I.2. Correlation Functions

One method to quantify coherent flow structure within the AN-MPCD fluid is to analyse the spatial correlation functions

$$C_{xy}(R) = \frac{\langle \boldsymbol{x}(\boldsymbol{r}_0;t) \cdot \boldsymbol{y}(\boldsymbol{r}_0 + R\hat{r};t)\rangle}{\langle \boldsymbol{x}(\boldsymbol{r}_0;t) \cdot \boldsymbol{y}(\boldsymbol{r}_0;t)\rangle}, \tag{SI 3}$$

between two fields x and y. These unspecified fields can be vector fields, such as the director or velocity fields $\boldsymbol{x}, \boldsymbol{y} \in \{\boldsymbol{n}, \boldsymbol{v}\}$, or scalar fields, such as density, flow speed, vorticity or nematic order parameter $x, y \in \{N_c, v, \omega, S\}$. The separation distance between two points in the fluid is R and $\hat{r}$ is the radial unit vector. The average is over all points in space and time.

Chiefly, we consider auto-correlation functions where $\boldsymbol{y} = \boldsymbol{x}$:

$$C_{xx}(R) = \frac{\langle \boldsymbol{x}(\boldsymbol{r}_0;t) \cdot \boldsymbol{x}(\boldsymbol{r}_0 + R\hat{r};t)\rangle}{\langle \boldsymbol{x}(\boldsymbol{r}_0;t) \cdot \boldsymbol{x}(\boldsymbol{r}_0;t)\rangle}. \tag{SI 4}$$

Since this work focuses on the hydrodynamics that results from activity, a thermal cutoff range $R_{k_\text{B}T}$ is applied. The thermal cutoff $R_{k_\text{B}T}$ is chosen to remove rapid thermal decorrelations that are present in the moderate-Péclet number MPCD method. For velocity correlations $C_{vv}(R)$ and director correlations $C_{nn}(R)$, the cutoff is $R_{k_\text{B}T} = 1$; whereas, $R_{k_\text{B}T} = 3$ for vorticity correlations $C_{\omega\omega}(R)$ due to the use of gradients when computing vorticity. Values $R \leq R_{k_\text{B}T}$ are removed, and the remaining correlation function is linearly interpolated from $R = R_{k_\text{B}T}$ to $R = 0$,

which renormalises the correlation function. However, if the renormalisation constant is too large then the correlation function is dominated by thermal effects, and consequently neglected.

Hydrodynamic correlations tend to initially decay and possess an anticorrelation well [24]. To measure active length scales from autocorrelation functions, the decorrelation length is determined by fitting an exponential decay to the small-R hydrodynamic region. The fit is performed for the range $C_{xx} > e^{-1} \cdot \lim_{R\to\infty} C_{xx}$.

Enstrophy spectra are employed when studying the spatial structure of traditional turbulence, and comparisons have been made to mesoscale active turbulence [25, 75]. For example, it has been found that Komogorov's universal $-5/3$ scaling exponent for inertial turbulence does not hold [25]. The enstrophy $\Omega = |\boldsymbol{\omega} \cdot \boldsymbol{\omega}|$ represents the magnitude of the vorticity $\boldsymbol{\omega} = \boldsymbol{\nabla} \times \boldsymbol{v}$. In this work, the enstrophy spectra are computed via the radial Fourier transform of the vorticity correlation function

$$E_{\Omega}\left(k\right) = \int_0^{\infty} C_{\omega\omega}\left(R\right) J_0(kR) R \, dR \tag{SI 5}$$

where J_0 is the Bessel function of the first kind with order zero.

I.3. Density Analysis

A common feature of active particle-based models is their tendency to exhibit large variations in local density [3, 17]. Nematic adaptations of the Vicsek model exhibit such *giant number fluctuations* [12, 17, 19]. These are also commonly observed in experimental and theoretical work on dry active-nematics [57, 76]. Furthermore, significant density gradients can occur in wet active nematic suspensions, including in filaments/motor proteins nematic films [27, 30], and in bacterial turbulence, which can exhibit both long-range nematic order and anomalous fluctuations [77]. Commonly assumed incompressibility conditions of hydrodynamic models should not be seen as rigorous experimental fact, but rather as a simplifying assumption, akin to the one-elastic constant assumption. As a particle-based algorithm for active nematics, AN-MPCD is expected to exhibit significant number fluctuations and, because the activity depends on the number of active agents (Eq. 7 for constant particle activity), the local density governs the local activity.

In the typical case of equilibrium systems, density distributions are Gaussian due to the *central limit theorem* [76]. However, continuous energy injection can lead to non-Gaussian distributions. Hence, a measure of a distribution's Gaussianity can be revealing. A non-Gaussianity measure (NGM) can be defined to be

$$\chi_{\mathrm{NGM}} = \frac{d}{d+2} \frac{\Delta r^4}{\left|\Delta r^2\right|^2} - 1 \tag{SI 6}$$

where $d = 2$ is the dimension and Δr^k is the k'th moment of the distribution [14]. When a distribution is purely Gaussian $\chi_{\mathrm{NGM}} = 0$. However, $\chi_{\mathrm{NGM}} > 0$ occurs when the tails of the distribution stretch, and $\chi_{\mathrm{NGM}} < 0$ occurs when the tails contract relative to normal. Physically, $\chi_{\mathrm{NGM}} \simeq 0$ holds for density distributions of systems in equilibrium, while active-particle models exhibit non-Gaussianity.

To quantify the fluctuations in density $\rho = \langle N_c \rangle / a^d$, consider how the standard deviation of density, σ_{N_c}, scales with the mean number density $\langle N_c \rangle$ [76]. In equilibrium, one expects

$$\sigma_{N_c} \sim \langle N_c \rangle^{\nu}, \tag{SI 7}$$

with the scaling $\nu = 1/2$ given by the central limit theorem. However, in systems of active particles, $\nu > 1/2$ is typical. As the scaling approaches $\nu \simeq 1$, an active system is said to exhibit giant number fluctuations. These fluctuations in density are predicted by theory [57], experiments [76] and observed in simulations [78]. The simulation domain is segmented into sub-domains in order to quantify these. By averaging over these sub-domains and computing the mean and standard deviations, the scaling ν is obtained.

II. MOVIE CAPTIONS

1. **Movie S1: Instabilities lead to defect pair creation and active turbulence.** Director field coloured by nematic order parameter S_c demonstrating defect unbinding. $\alpha = 0.03$, $\ell_{\mathrm{sys}} = 30$, simulation length is $1000\delta t$, and each frame is $5\delta t$ apart. Each line segment corresponds to the director in one MPCD cell and no smoothing has been applied to the images.

2. **Movie S2: Instabilities lead to vorticity bands and active turbulence.** Velocity field $\boldsymbol{v}$ (black arrows) coloured underneath by scalar value of vorticity ω, corresponding to Movie 1.

3. <u>**Movie S3:**</u> **For sufficiently low activity, injected energy can be absorbed by the thermostat and the system remains globally ordered.** Director field coloured by nematic order parameter S_c for $\alpha < \alpha_{\text{eq}}$; activity value $\alpha = 0.0008$. The system size is $\ell_{\text{sys}} = 100$ and duration $2500\delta t$ following a $2000\delta t$ warmup. Each frame is $5\delta t$ apart. Each line segment corresponds to the director in every other MPCD cell, and this is true for directors and velocity arrows in Movies 3-16.

4. <u>**Movie S4:**</u> **For the low activity regime, kink walls in the director field form and persistently existence with a characteristic separation length.** Director field coloured by nematic order parameter S_c for $\alpha_{\text{eq}} < \alpha < \alpha_{\text{turb}}$; activity value $\alpha = 0.008$. All other parameters are the same as Movie 3.

5. <u>**Movie S5:**</u> **Fully developed active turbulence.** Director field coloured by nematic order parameter S_c for $\alpha_{\text{turb}} < \alpha < \alpha_{\dagger}$; activity value $\alpha = 0.08$. All other parameters are the same as Movie 3.

6. <u>**Movie S6:**</u> **Fully developed active turbulence with a steady-state population of defects.** Movie 5 with computed defect positions overlaid. $-1/2$ defects are marked as blue trifolds, and $+1/2$ defects are marked with red arrows.

7. <u>**Movie S7:**</u> **Velocity field corresponding to Movie 3.** Velocity field coloured by speed $|\boldsymbol{v}|$ with activity $\alpha = 0.0008$.

8. <u>**Movie S8:**</u> **Velocity field corresponding to Movie 4.** Velocity field coloured by speed $|\boldsymbol{v}|$ with activity $\alpha = 0.008$.

9. <u>**Movie S9:**</u> **Velocity field corresponding to Movie 5.** Velocity field coloured by speed $|\boldsymbol{v}|$ with activity $\alpha = 0.08$.

10. <u>**Movie S10:**</u> **Movie 9 with computed defect positions overlaid.** $-1/2$ defects are marked as blue trifolds, and $+1/2$ defects are marked with red arrows.

11. <u>**Movie S11:**</u> **Density field corresponding to Movie 5.** Number density field $N_C/N_C^{\max}$, where $N_C^{\max}$ is the instantaneous maximum cell population. The simulation in this movie is the same as Movie 5 for $\alpha = 0.08$, with identical parameters and frame times.

12. <u>**Movie S12:**</u> **High-density bands within a sparse nematic gas at high activities $\alpha \gtrsim \alpha_{\dagger}$.** Number density field $N_C/N_C^{\max}$ for $\alpha_{\dagger} < \alpha$; activity value $\alpha = 0.3$. All other parameters are the same as Movie 11

13. <u>**Movie S13:**</u> **Director field coloured corresponding to Movie 12.** Director field coloured by nematic order parameter S_c with activity $\alpha = 0.3$.

14. <u>**Movie S14:**</u> **Movie 13 with computed defect positions overlaid.** $-1/2$ defects are marked as blue trifolds, and $+1/2$ defects are marked with red arrows.

15. <u>**Movie S15:**</u> **Velocity field corresponding to Movie 12.** Velocity field coloured by speed $|\boldsymbol{v}|$ with activity $\alpha = 0.3$.

16. <u>**Movie S16:**</u> **Movie 15 with computed defect positions overlaid.** $-1/2$ defects are marked as blue trifolds, and $+1/2$ defects are marked with red arrows.

III. SUPPLEMENTARY FIGURES

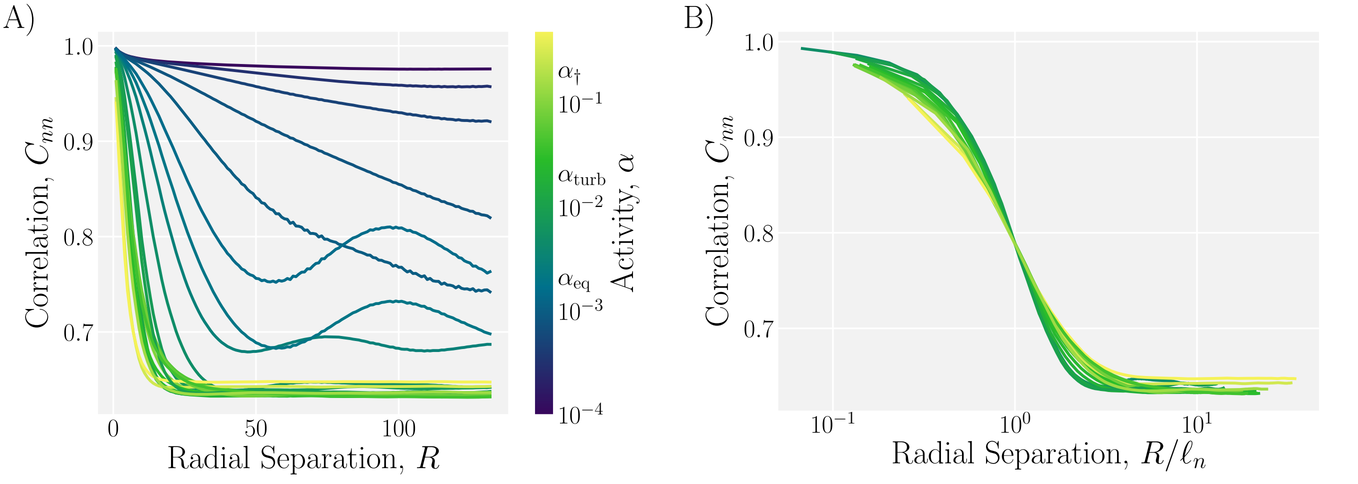

Fig. S1. **Director spatial structure.** **(A)** Director-director spatial correlation function $C_{nn}(R)$. **(B)** Director-director spatial correlation function rescaled by nematic decorrelation length ℓ_n.

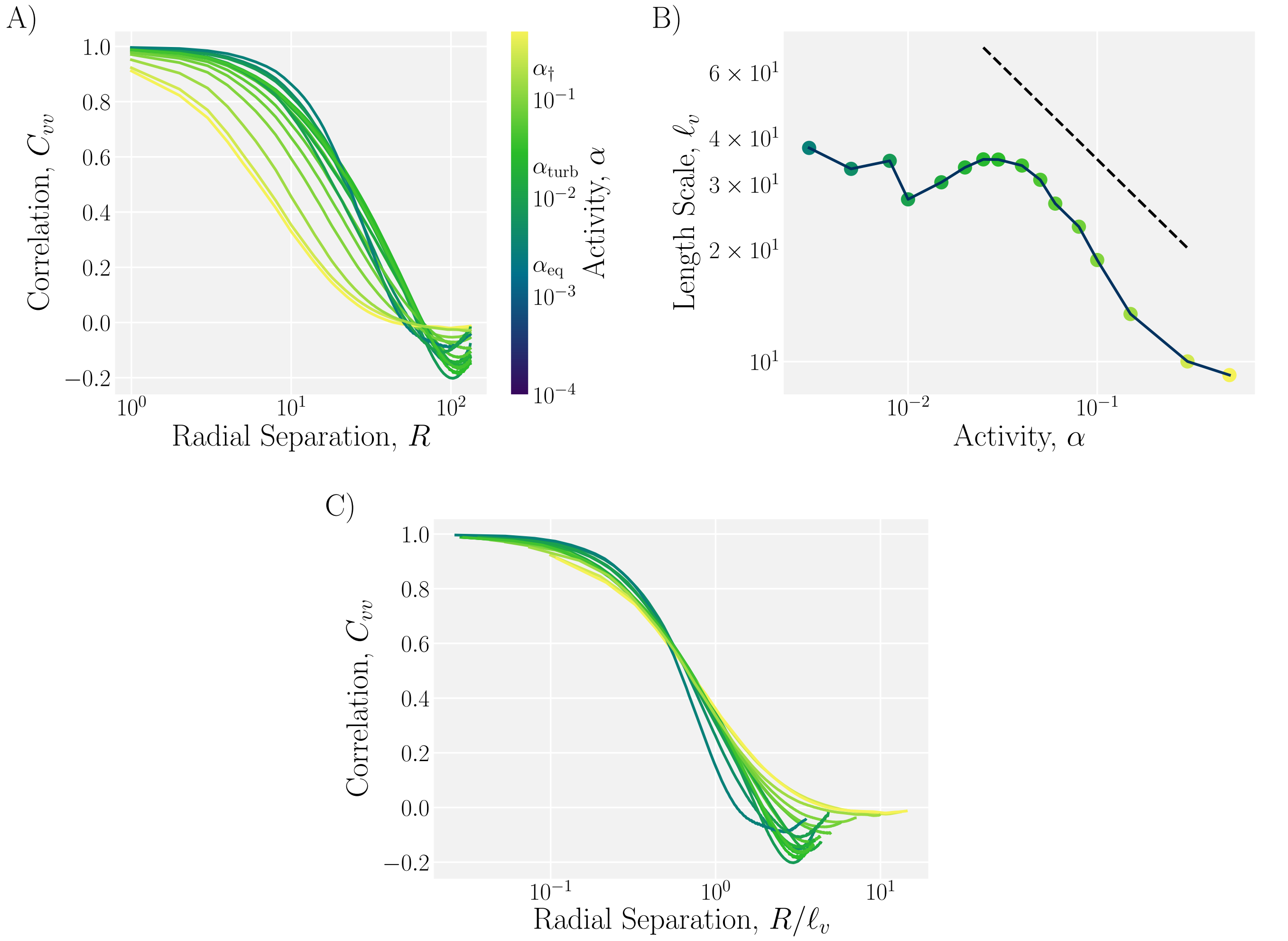

Fig. S2. **Spatial structure of active turbulence.** **(A)** Radial hydrodynamic autocorrelation functions of velocity for various activities above α_{eq}. **(B)** The velocity length scales as computed from the correlation functions in fig. S2A. The dashed line indicates a scaling of $\ell_v \sim \alpha^{\mu}$ with $\mu = -1/2$. **(C)** Scaling the correlation functions in fig. S2A by the corresponding length scale in fig. S2B.

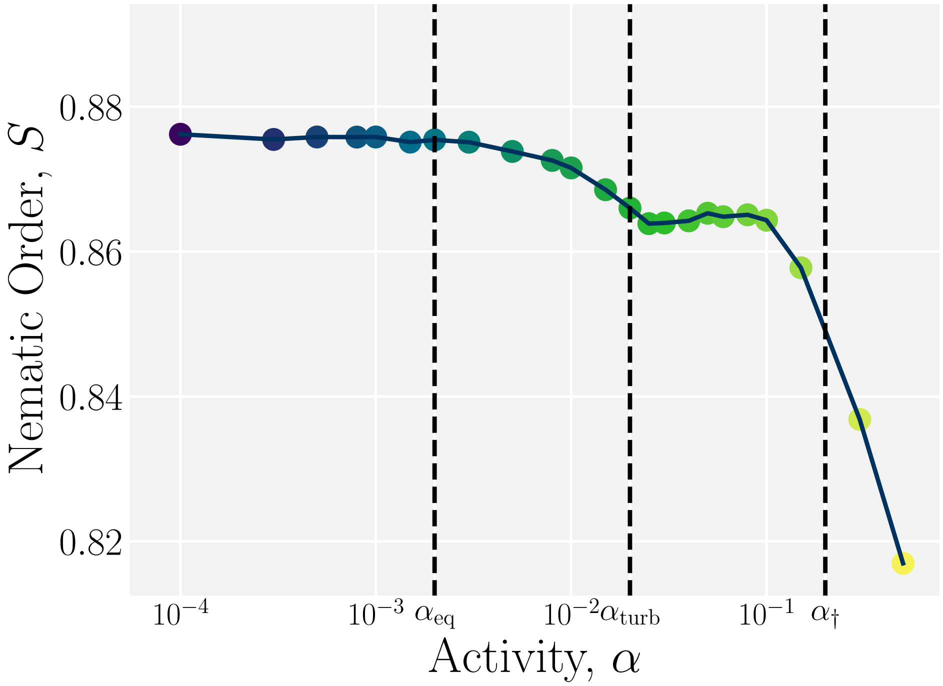

Fig. S3. **Average nematic order in AN-MPCD.** Four regimes are visible, corresponding to (i) $\alpha \lesssim \alpha_{\text{eq}}$ nematic behaviour, (ii) $\alpha_{\text{eq}} \lesssim \alpha \lesssim \alpha_{\text{turb}}$ onset of active effects, (iii) $\alpha_{\text{turb}} \lesssim \alpha_{\dagger}$ active turbulence, (iv) $\alpha_{\dagger} \lesssim \alpha$ active turbulence scalings no longer achieved and local order plummets.

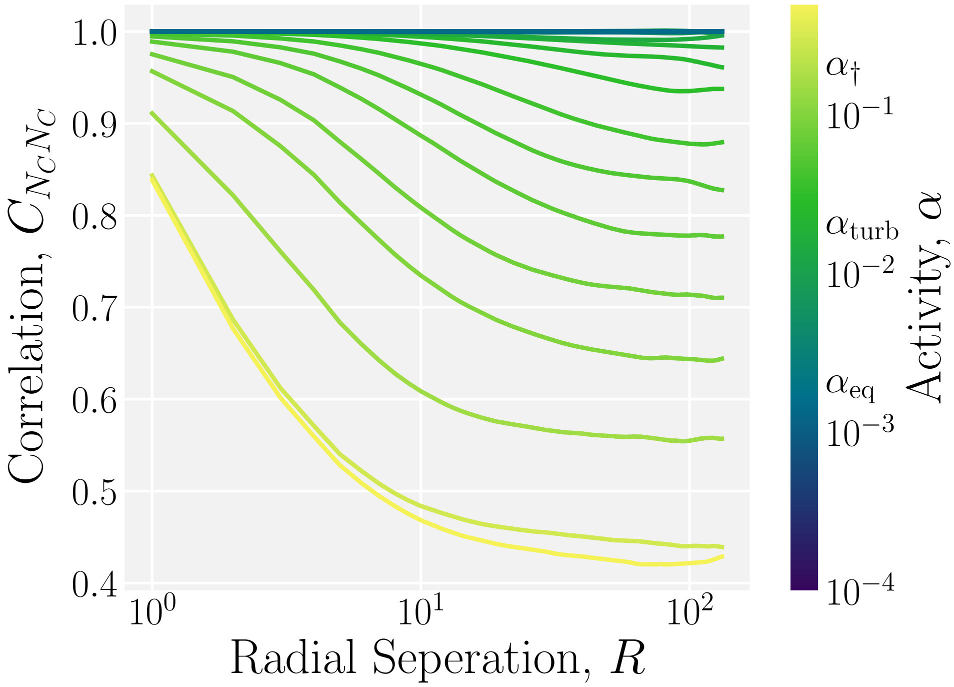

Fig. S4. **Density correlations in active MPCD.** Radial hydrodynamic autocorrelation functions of cell density N_c for activities α.

REFERENCES AND NOTES

1. J. Toner, Y. Tu, S. Ramaswamy, Hydrodynamics and phases of flocks. *Ann. Phys. Rehabil. Med.* **318**, 170–244 (2005).

2. M. C. Marchetti, J.-F. Joanny, S. Ramaswamy, T. B. Liverpool, J. Prost, M. Rao, R. A. Simha, Hydrodynamics of soft active matter. *Rev. Mod. Phys.* **85**, 1143–1189 (2013).

3. M. Bär, R. Großmann, S. Heidenreich, F. Peruani, Self-propelled rods: Insights and perspectives for active matter. *Annu. Rev. Condens. Matter Phys.* **11**, 441–466 (2020).

4. J. Elgeti, R. G. Winkler, G. Gompper, Physics of microswimmers—Single particle motion and collective behavior: A review. *Rep. Prog. Phys.* **78**, 056601 (2015).

5. Z. You, D. J. G. Pearce, A. Sengupta, L. Giomi, Geometry and mechanics of microdomains in growing bacterial colonies. *Phys. Rev. X* **8**, 031065 (2018).

6. L. Atia, D. Bi, Y. Sharma, J. A. Mitchel, B. Gweon, S. A. Koehler, S. J. DeCamp, B. Lan, J. H. Kim, R. Hirsch, A. F. Pegoraro, K. H. Lee, J. R. Starr, D. A. Weitz, A. C. Martin, J.-A. Park, J. P. Butler, J. J. Fredberg, Geometric constraints during epithelial jamming. *Nat. Phys.* **14**, 613–620 (2018).

7. A. Mongera, P. Rowghanian, H. J. Gustafson, E. Shelton, D. A. Kealhofer, E. K. Carn, F. Serwane, A. A. Lucio, J. Giammona, O. Campàs, A fluid-to-solid jamming transition underlies vertebrate body axis elongation. *Nature* **561**, 401–405 (2018).

8. T. Sanchez, D. T. Chen, S. J. DeCamp, M. Heymann, Z. Dogic, Spontaneous motion in hierarchically assembled active matter. *Nature* **491**, 431–434 (2012).

9. D. Nishiguchi, K. H. Nagai, H. Chaté, M. Sano, Long-range nematic order and anomalous fluctuations in suspensions of swimming filamentous bacteria. *Phys. Rev. E* **95**, 020601 (2017).

10. D. Dell’Arciprete, M. Blow, A. Brown, F. Farrell, J. S. Lintuvuori, A. McVey, D. Marenduzzo, W. C. Poon, A growing bacterial colony in two dimensions as an active nematic. *Nat. Commun.* **9**, 4190 (2018).

11. G. Duclos, S. Garcia, H. G. Yevick, P. Silberzan, Perfect nematic order in confined monolayers of spindle-shaped cells. *Soft Matter* **10**, 2346–2353 (2014).

12. F. Ginelli, F. Peruani, M. Bär, H. Chaté, Large-scale collective properties of self-propelled rods. *Phys. Rev. Lett.* **104**, 184502 (2010).

13. A. Baskaran, M. C. Marchetti, Self-regulation in self-propelled nematic fluids. *Eur. Phys. J. E Soft Matter* **35**, 95 (2012).

14. A. M. Nagel, M. Greenberg, T. N. Shendruk, H. W. de Haan, Collective dynamics of model pili-based twitcher-mode bacilliforms. *Sci. Rep.* **10**, 10747 (2020).

15. T. Gao, M. D. Betterton, A.-S. Jhang, M. J. Shelley, Analytical structure, dynamics, and coarse graining of a kinetic model of an active fluid. *Phys. Rev. Fluids* **2**, 093302 (2017).

16. A. Zöttl, J. M. Yeomans, Enhanced bacterial swimming speeds in macromolecular polymer solutions. *Nat. Phys.* **15**, 554–558 (2019).

17. H. Chaté, F. Ginelli, R. Montagne, Simple model for active nematics: Quasi-long-range order and giant fluctuations. *Phys. Rev. Lett.* **96**, 180602 (2006).

18. S. Ngo, F. Ginelli, H. Chaté, Competing ferromagnetic and nematic alignment in self-propelled polar particles. *Phys. Rev. E* **86**, 050101 (2012).

19. A. Patelli, I. Djafer-Cherif, I. S. Aranson, E. Bertin, H. Chaté, Understanding dense active nematics from microscopic models. *Phys. Rev. Lett.* **123**, 258001 (2019).

20. C. Valeriani, M. Li, J. Novosel, J. Arlt, D. Marenduzzo, Colloids in a bacterial bath: Simulations and experiments. *Soft Matter* **7**, 5228 (2011).

21. L. Giomi, M. J. Bowick, P. Mishra, R. Sknepnek, M. C. Marchetti, Defect dynamics in active nematics. *Philos. Trans. A Math. Phys. Eng. Sci.* **372**, 20130365 (2014).

22. S. P. Thampi, R. Golestanian, J. M. Yeomans, Instabilities and topological defects in active nematics. *Europhys. Lett.* **105**, 18001–18001 (2014a).

23. H. H. Wensink, J. Dunkel, S. Heidenreich, K. Drescher, R. E. Goldstein, H. Löwen, J. M. Yeomans, Mesoscale turbulence in living fluids. *Proc. Natl. Acad. Sci.* **109**, 14308–14313 (2012).

24. S. P. Thampi, R. Golestanian, J. M. Yeomans, Vorticity, defects and correlations in active turbulence. *Philos. Trans. A Math. Phys. Eng. Sci.* **372**, 20130366 (2014b).

25. R. Alert, J.-F. Joanny, J. Casademunt, Universal scaling of active nematic turbulence. *Nat. Phys.* **16**, 682–688 (2020).

26. R. Alert, J. Casademunt, J.-F. Joanny, Active turbulence. *Annu. Rev. Condens. Matter Phys.* **13**, 143–170 (2022).

27. A. Opathalage, M. M. Norton, M. P. N. Juniper, B. Langeslay, S. A. Aghvami, S. Fraden, Z. Dogic, Self-organized dynamics and the transition to turbulence of confined active nematics. *Proc. Natl. Acad. Sci.* **116**, 4788–4797 (2019).

28. A. Chardac, S. Shankar, M. C. Marchetti, D. Bartolo, Emergence of dynamic vortex glasses in disordered polar active fluids. *Proc. Natl. Acad. Sci.* **118**, e2018218118 (2021).

29. A. Sokolov, A. Mozaffari, R. Zhang, J. J. De Pablo, A. Snezhko, Emergence of radial tree of bend stripes in active nematics. *Phys. Rev. X* **9**, 031014 (2019).

30. K. Thijssen, D. A. Khaladj, S. A. Aghvami, M. A. Gharbi, S. Fraden, J. M. Yeomans, L. S. Hirst, T. N. Shendruk, Submersed micropatterned structures control active nematic flow, topology, and concentration. *Proc. Natl. Acad. Sci. U.S.A.* **118**, e2106038118 (2021).

31. R. R. Keogh, S. Chandragiri, B. Loewe, T. Ala-Nissila, S. P. Thampi, T. N. Shendruk, Helical flow states in active nematics. *Phys. Rev. E* **106**, L012602 (2022).

32. B. Loewe, T. N. Shendruk, Passive Janus particles are self-propelled in active nematics. *New J. Phys.* **24**, 012001 (2022).

33. G. Foffano, J. Lintuvuori, K. Stratford, M. Cates, D. Marenduzzo, Colloids in active fluids: Anomalous microrheology and negative drag. *Phys. Rev. Lett.* **109**, 028103 (2012).

34. D. P. Rivas, T. N. Shendruk, R. R. Henry, D. H. Reich, R. L. Leheny, Driven topological transitions in active nematic films. *Soft Matter* **16**, 9331–9338 (2020).

35. A. Lagarde, N. Dagès, T. Nemoto, V. Démery, D. Bartolo, T. Gibaud, Colloidal transport in bacteria suspensions: From bacteria collision to anomalous and enhanced diffusion. *Soft Matter* **16**, 7503–7512 (2020).

36. S. K. Anand, S. P. Singh, Conformation and dynamics of a self-avoiding active flexible polymer. *Phys. Rev. E* **101**, 030501 (2020).

37. M. Shafiei Aporvari, M. Utkur, E. U. Saritas, G. Volpe, J. Stenhammar, Anisotropic dynamics of a self-assembled colloidal chain in an active bath. *Soft Matter* **16**, 5609–5614 (2020).

38. R. G. Winkler, J. Elgeti, G. Gompper, Active polymers—Emergent conformational and dynamical properties: A brief review. *J. Physical Soc. Japan* **86**, 101014 (2017).

39. J. C. Llahí, A. Martín-Gómez, G. Gompper, R. G. Winkler, Simulating wet active polymers by multiparticle collision dynamics. *Phys. Rev. E* **105**, 015310 (2022).

40. E. Westphal, S. Singh, C.-C. Huang, G. Gompper, R. Winkler, Multiparticle collision dynamics: GPU accelerated particle-based mesoscale hydrodynamic simulations. *Comput. Phys. Commun.* **185**, 495–503 (2014).

41. M. P. Howard, A. Z. Panagiotopoulos, A. Nikoubashman, Efficient mesoscale hydrodynamics: Multi-particle collision dynamics with massively parallel GPU acceleration. *Comput. Phys. Commun.* **230**, 10–20 (2018).

42. A. Malevanets, R. Kapral, Mesoscopic model for solvent dynamics. *J. Chem. Phys.* **110**, 8605–8613 (1999).

43. G. Gompper, T. Ihle, D. Kroll, R. Winkler, Multi-particle collision dynamics: A particle-based mesoscale simulation approach to the hydrodynamics of complex fluids, in *Advanced Computer Simulation Approaches for Soft Matter Sciences III* (Springer, 2009), pp. 1–87.

44. A. W. Zantop, H. Stark, Multi-particle collision dynamics with a non-ideal equation of state. I. *J. Chem. Phys.* **154**, 024105 (2021).

45. M. James, D. A. Suchla, J. Dunkel, M. Wilczek, Emergence and melting of active vortex crystals. *Nat. Commun.* **12**, 5630 (2021).

46. M. P. Howard, A. Nikoubashman, J. C. Palmer, Modeling hydrodynamic interactions in soft materials with multiparticle collision dynamics. *Curr. Opin. Chem. Eng.* **23**, 34–43 (2019).

47. H. Noguchi, N. Kikuchi, G. Gompper, Particle-based mesoscale hydrodynamic techniques. *Europhys. Lett.* **78**, 10005 (2007).

48. T. N. Shendruk, J. M. Yeomans, Multi-particle collision dynamics algorithm for nematic fluids. *Soft Matter* **11**, 5101–5110 (2015).

49. D. Reyes-Arango, J. Quintana-H., J. C. Armas-Pérez, H. Híjar, Defects around nanocolloids in nematic solvents simulated by multi-particle collision dynamics. *Physica A* **547**, 123862 (2020).

50. K.-W. Lee, M. G. Mazza, Stochastic rotation dynamics for nematic liquid crystals. *J. Chem. Phys.* **142**, 164110 (2015).

51. S. Mandal, M. G. Mazza, Multiparticle collision dynamics for tensorial nematodynamics. *Phys. Rev. E* **99**, 063319 (2019).

52. H. Híjar, R. Halver, G. Sutmann, Spontaneous fluctuations in mesoscopic simulations of nematic liquid crystals. *Fluctuat. Noise Lett.* **18**, 1950011 (2019).

53. H. Híjar, Hydrodynamic correlations in isotropic fluids and liquid crystals simulated by multi-particle collision dynamics. arXiv:1903.11474 (2019).

54. K.-W. Lee, T. Pöschel, Electroconvection of pure nematic liquid crystals without free charge carriers. *Soft Matter* **13**, 8816–8823 (2017).

55. H. Híjar, Dynamics of defects around anisotropic particles in nematic liquid crystals under shear. *Phys. Rev. E* **102**, 062705 (2020).

56. S. Mandal, M. G. Mazza, Multiparticle collision dynamics simulations of a squirmer in a nematic fluid. *Europ. Phys. J. E* **44**, 64 (2021).

57. S. Ramaswamy, R. A. Simha, J. Toner, Active nematics on a substrate: Giant number fluctuations and long-time tails. *Europhys. Lett.* **62**, 196–202 (2003).

58. D. Saintillan, Rheology of active fluids. *Annu. Rev. Fluid Mech.* **50**, 563 (2018).

59. L. M. Lemma, S. J. DeCamp, Z. You, L. Giomi, Z. Dogic, Statistical properties of autonomous flows in 2D active nematics. *Soft Matter* **15**, 3264–3272 (2019).

60. A. Doostmohammadi, M. F. Adamer, S. P. Thampi, J. M. Yeomans, Stabilization of active matter by flow-vortex lattices and defect ordering. *Nat. Commun.* **7**, 10557 (2016).

61. R. A. Simha, S. Ramaswamy, Statistical hydrodynamics of ordered suspensions of self-propelled particles: Waves, giant number fluctuations and instabilities. *Physica A* **306**, 262–269 (2002).

62. R. Voituriez, J.-F. Joanny, J. Prost, Spontaneous flow transition in active polar gels. *Europhys. Lett.* **70**, 404–410 (2005).

63. S. Shankar, S. Ramaswamy, M. C. Marchetti, M. J. Bowick, Defect unbinding in active nematics. *Phys. Rev. Lett.* **121**, 108002 (2018).

64. K. Binder, P. Virnau, Phase transitions and phase coexistence: Equilibrium systems versus externally driven or active systems - some perspectives. *Soft Mater.* **19**, 267–285 (2021).

65. B. Mahault, F. Ginelli, H. Chaté, Quantitative assessment of the toner and tu theory of polar flocks. *Phys. Rev. Lett.* **123**, 218001 (2019).

66. L. B. Weiss, C. N. Likos, A. Nikoubashman, Spatial demixing of ring and chain polymers in pressure-driven flow. *Macromolecules* **52**, 7858–7869 (2019).

67. Z. Wang, R. Wang, Y. Lu, L. An, A.-C. Shi, Z.-G. Wang, Mechanisms of flow-induced polymer translocation. *Macromolecules* **55**, 3602–3612 (2022).
